# Characterization of relativistic electron–positron beams produced with laser-accelerated GeV electrons

**DOI:** 10.1038/s41598-023-27617-0

**Published:** 2023-01-06

**Authors:** Hoon Song, Chul Min Kim, Junho Won, Jaehyun Song, Seongmin Lee, Chang-Mo Ryu, Woosuk Bang, Chang Hee Nam

**Affiliations:** 1 Center for Relativistic Laser Science, Institute for Basic Science (IBS), Gwangju, 61005 Korea; 2 Department of Physics and Photon Science, GIST, Gwangju, 61005 Korea; 3 Advanced Photonics Research Institute, GIST, Gwangju, 61005 Korea

**Keywords:** Physics, Plasma physics, Laser-produced plasmas

## Abstract

The characterization of an electron–positron beam generated from the interaction of a multi-GeV electron beam with a lead plate is performed using GEANT4 simulations. The dependence of the positron beam size on driver electron beam energy and lead converter thickness is investigated in detail. A pancake-like positron beam structure is generated with a monoenergetic multi-GeV driver electron beam, with the results indicating that a 5 GeV driver electron beam with 1 nC charge can generate a positron beam with a density of 10^15^–10^16^ cm^−3^ at one radiation length of lead. In addition, we find that electron–positron beams generated using above-GeV electron beams have neutralities greater than 0.3 at one radiation length of lead, whereas neutralities of 0.2 are observed when using a 200 MeV electron beam. The possibility of observing plasma instabilities in experiments is also examined by comparing the plasma skin depth with the electron–positron beam size. A quasi-neutral electron–positron plasma can be produced in the interaction between a 1 nC, 5 GeV electron beam and lead with a thickness of five radiation lengths. Our findings will aid in analyzing and interpreting laser-produced electron–positron plasma for laboratory astrophysics research.

## Introduction

Recent research interests in the generation of relativistic positron beams using ultra-intense lasers stem from the prospect of replicating astrophysical phenomena in a laboratory^1–3^. In particular, electron–positron pairs are ubiquitous in energetic astrophysical objects such as quasars^4^ and other galactic centers^5^. Moreover, electromagnetic instability, such as Weibel instabilities in electron–positron plasmas are believed to play a crucial role in the formation of relativistic collisionless shocks in gamma-ray bursts^6,7^.

There are two different experimental techniques for replicating astrophysical phenomena involving electron–positron plasmas with high intensity lasers. First, an intense laser pulse (> 10^18^ W/cm^2^) can irradiate a high-*Z* material directly, causing electrons on the front surface of the material to accelerate via the $\overrightarrow{J}\times \overrightarrow{B}$ mechanism and generate positrons through a combination of bremsstrahlung and Bethe–Heitler processes. This technique is straightforward to implement and has the potential to generate a large number of positrons^8,9^, but it typically generates low energy (< 100 MeV) electrons and positrons with a large divergence (> 100 mrad).

The second technique involves the production of an energetic driver electron beam by accelerating electrons to high kinetic energies in a plasma using the laser wakefield acceleration (LWFA) mechanism and then using the resulting LWFA electron beam to irradiate a high-Z material. Although this technique is based on the same physical processes (bremsstrahlung and Bethe–Heitler processes) for generating positrons, it can generate positrons with highly relativistic energies (> 100 MeV) with a low divergence because the initial LWFA electron beam is nearly collimated. With the recent advances in laser technology^10^, electrons with energies of several GeV are achievable using the LWFA mechanism^11–14^, thereby allowing the generation of ultra-relativistic positron beams in a laboratory. For example, using a GeV-scale LWFA electron beam it is possible to produce a near-GeV electron–positron beam^15^. In this regard, we examined situations in which a multi-GeV driver electron beam collided with a lead plate and analyzed the structure of the generated high-energy positron beams.

Our theoretical study investigated the electron–positron beam structure assuming that a monoenergetic electron beam with no divergence was incident on a lead converter. This allows us to predict the size of the positron beam in positron-beam generation experiments. In experiments, the driver electron beam produced by the LWFA mechanism has both an energy spread and a finite source size. This makes it difficult to calculate the spectrum and size of the generated positrons. We also considered the conditions under which the generated electron–positron beam could be called a plasma. We examined the density and quasi-neutrality of electron–positron beams and determined whether the beam size was greater than the plasma skin depth, which is the criterion for observing the corresponding instability in an experiment.

The remainder of this paper is organized as follows. The simulation conditions are described in “Simulation method”. In “Positron beam structures”, we define the size of the positron beam generated by a monoenergetic driver electron beam and examine essential beam characteristics of generated positron beams—such as size, divergence, and yield—for various driver electron energies and lead converter thicknesses. Furthermore, we examine the dependence of lead converter thicknesses on the positron beam size. Finally, in “Neutrality, density, and size”, we demonstrate the generation of a high-density pair plasma using a GeV LWFA driver electron beam with a lead converter by calculating the neutrality, density, and skin depth conditions of generated electron–positron beam.

## Simulation method

We studied the passage of high-energy electrons through a lead converter of different thicknesses using the Monte Carlo simulation code, GEANT4^16^. In each simulation run, a total of 10^6^ computational monoenergetic electrons propagated along the *x*-axis with initial kinetic energies of 200 MeV, 500 MeV, 1 GeV, 2 GeV, 5 GeV, and 10 GeV. The physics package, QGSP_BERT, is used in our simulations, which includes the energy loss processes of electrons and positrons such as bremsstrahlung, Coulomb scattering, and ionization. This package does not include the electromagnetic field effects or the collective behavior of the electron and positron beams. Generated bremsstrahlung photons are subject to pair-production via the Bethe–Heitler process. The photon interaction such as Compton scattering and photoelectron generation is also included in this package, and the range cutoff value was set to 1 mm in our simulations. In these simulations, pencil-beam electrons with initial divergences of zero were used, which is a valid assumption for driver electron beams with divergences that are sufficiently small compared with that of the generated electron–positron beam in an experiment. This is the case for the LWFA driver electron beam, which has a small divergence (< 5 mrad) compared with that of the generated electron–positron beam (> 100 mrad)^17^.

After each simulation run, the phase-space information of generated positrons with kinetic energies larger than 1 MeV was recorded on the back surface of the lead converter. Then, we advanced all the particles’ positions until a specific time, $({t\cdot L}_{rad}/c+3 \mathrm{ps}),$ where $t$ is the lead converter thickness normalized by the radiation length^18,19^, ${L}_{rad}$, and $c$ is the speed of light. This way, we were able to take snapshots of the generated positrons after they exited the converters. The timing of the snapshot was chosen as the sum of the time required for a photon to travel across the given converter thickness in vacuum plus extra three picoseconds to allow for most of the positrons to exit the converter. In our simulations, lead was used as a high-Z converter. Lead converters with thicknesses of 0.1*L*_*rad*_, 0.2*L*_*rad*_, 0.3*L*_*rad*_, 0.4*L*_*rad*_, …, 2.7*L*_*rad*_, 2.8*L*_*rad*_, 2.9*L*_*rad*_, 3.0*L*_*rad*_, 3.3*L*_*rad*_, 3.5*L*_*rad*_, 3.7*L*_*rad*_, 4.0*L*_*rad*_, 4.5*L*_*rad*_, and 5.0*L*_*rad*_ were used in our simulations, where the radiation length for lead is approximately 5.6 mm.

## Positron beam structures

**Figure 1:**
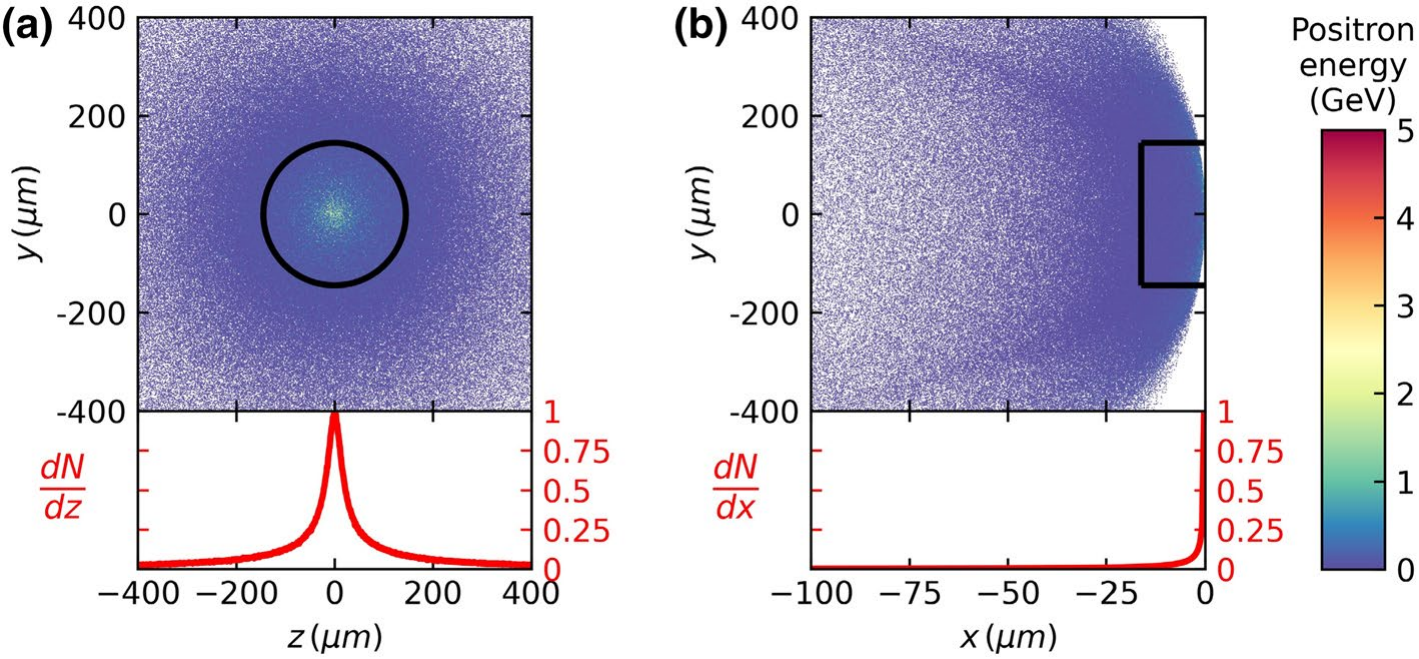
(**a**) Transversal distribution of the generated positrons. Each dot represents a positron and its color represents the kinetic energy of the positron. The normalized positron number density in the radial direction is shown as a red curve. (**b**) Longitudinal distribution of the generated positrons. The black rectangle represents the dimensions of the positron beam. The thickness of the box is set as one standard deviation, δ*x*, of the positrons along the *x-*axis within ${r}_{median}$. The red curve represents the normalized positron number density within *r*_*median*_ along the *x*-axis normalized by its peak value at *x* = 0, $\frac{dN}{dx}$ (right vertical axis). The driver electron energy is 5 GeV, and the lead converter thickness is *L*_*rad*_.

We investigated the distribution of the generated positrons in real space. Although the GEANT4 code did not show how the generated positrons were distributed in space after they exited the converter, we have successfully visualized the spatial distribution of positrons through our post-processing procedure. We find that a relativistic monoenergetic electron pencil-beam produces a pancake-shaped positron beam. Figure 1a,b illustrate the spatial distributions of the generated positrons when the driver electron energy is 5 GeV and the thickness of the lead converter is *L*_*rad*_. We have post-processed the phase-space information of the generated positrons recorded on the back surface of the lead converter so that we could visualize the generated positrons in real space after they exited the converter. We let all the recorded positrons propagate freely in space until ${T}_{0}=({t\cdot L}_{rad}/c+3 \mathrm{ps})$. Figure 1a shows the two-dimensional spatial distribution of positrons looking from the propagation direction at time ${T}_{0}$ and Fig. 1b illustrates the spatial distribution of positrons looking from the side at ${T}_{0}$. Note that an arc structure is visible in Fig. 1b, which we attribute to low-energy (< 50 MeV) positrons. (See Supplementary Figs. S1 and S2 for more details.) Each dot in Fig. 1a,b represents the position of a positron with its color representing the kinetic energy of the corresponding positron. The majority of the positrons have kinetic energies far less than the initial electron beam energy of 5 GeV, which is consistent with the typical Bethe–Heitler positron energy spectrum.

Figure 1a shows the transversal distribution of positrons. The generated positrons are distributed symmetrically along the propagation axis in Fig. 1a,b because we have used pencil-beam electrons as the driver electron beam in our simulations. Since the positron distribution is symmetrical along the propagation axis, the generated positrons can be represented using a cylindrical volume. In order to determine the appropriate volume that can represent the whole generated positrons, we define the radial size and longitudinal size of the positron beam. Hereafter, we will use the term “positron beam” to refer to the positron bunch within the cylindrical volume, shown as the black rectangle in Fig. 1b. We define the radius of the positron beam, *r*_*median*_, as the radius within which 50% of the generated positrons are present^20^. In Fig. 1a, the black circle represents *r*_*median*_ (= 145 $\upmu$m). The red curve represents the positron number density along the *z*-axis normalized by its peak value at *z* = 0, $\frac{dN}{dz}$. Figure 1b shows the longitudinal distribution of the generated positrons. We define the longitudinal thickness of the positron beam, δ*x*, as the standard deviation of the longitudinal positions of the positrons within *r*_*median*_ from the propagation axis. We find that about 80% of the positrons inside *r*_*median*_ are concentrated within δ*x* as can be estimated from the red curve in Fig. 1b, which illustrates the positron number density along the longitudinal direction within *r*_*median*_. The size of the positron beam is shown as a black rectangle in Fig. 1b, which has a thickness of δ*x* and a length of 2*r*_*median*_. In Fig. 1b, the driver electron beam propagates along the *x*-axis (= longitudinal direction), and the vertical axis represents one of the transverse directions. The longitudinal position of light traveling without divergence is set to zero.

The thickness (= δ*x*) of the positron beam turns out to be about 16 μm in Fig. 1b, which is much smaller compared with the diameter (= 2*r*_*median*_ = 290 µm) of the beam. The small beam thickness results from the small longitudinal velocity differences among the generated positrons. For example, the velocity difference between 5 GeV  and 10 MeV positrons is only approximately 10^–3^ *c*, which corresponds to a longitudinal position difference of 8 μm after a lead converter of thickness ${L}_{rad}$.

Although the defined volume occupies only a small portion of the whole beam, the small cylindrical volume contains approximately 40% (= 50% $\times$ ~ 80%) of all the generated positrons. Furthermore, our simulation results indicate that the sum of the positron energies in the volume accounts for approximately 90% of the total positron energy in Fig. 1a,b. To justify our definition for the size of the “positron beam”, we calculated the energy content of positron beams for different driver electron energies and lead converter thicknesses. As the driver electron energy decreases and the lead thickness increases, the energy content of the positrons inside the volume decreases. Interestingly, even at the lowest driver electron energy of 200 MeV and 5*L*_*rad*_, the energy contained inside the volume still exceeds 50% of the total positron energy. In summary, about 40% of total positrons are inside the volume carrying over 50% of the total positron energy, which justifies our definition of the positron beam.

**Figure 2:**
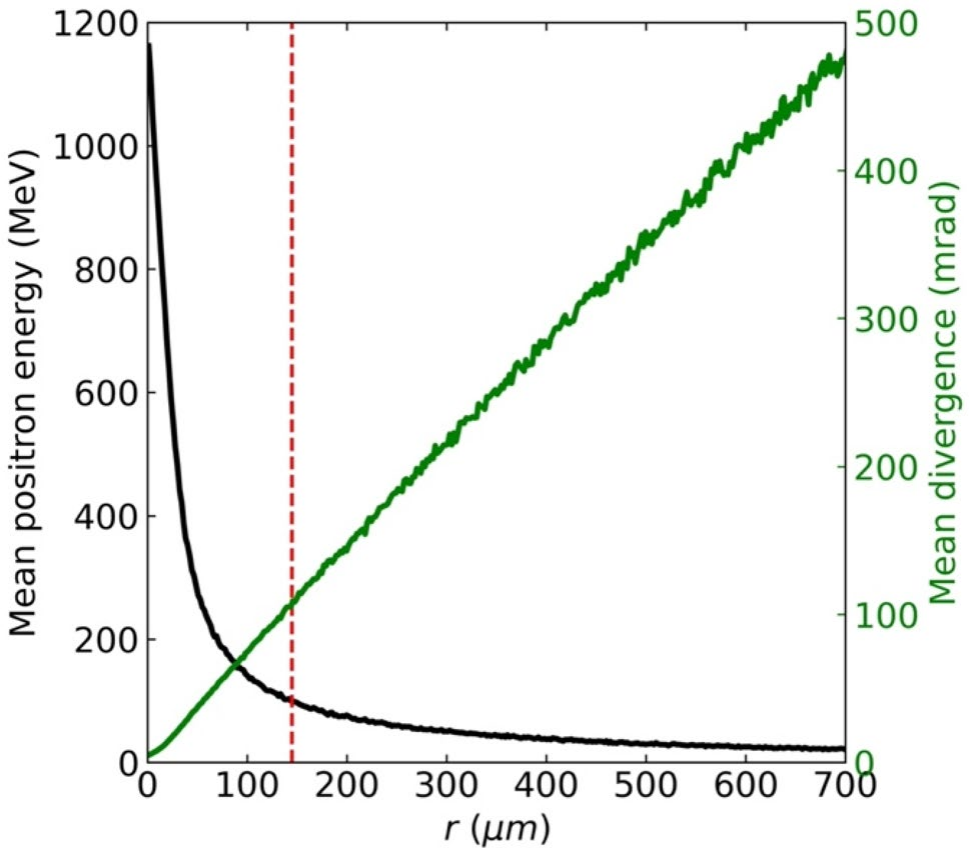
Radial dependence of positron beam characteristics. The mean positron energy at *r*, $<E\left(r\right)>$, is shown as a black curve. The dashed red line represents *r*_*median*_, defined in Fig. 1. The mean divergence of positron at *r*, $<\theta \left(r\right)>$, is shown as a green line. The driver electron energy is 5 GeV, and the lead converter thickness is *L*_*rad*_.

Figure 2 illustrates the radial dependence of the positron energy and divergence when a 5 GeV driver electron beam is incident on a lead converter of thickness *L*_*rad*_. Low-energy positrons are distributed toward the exterior (transverse direction) because particles with lower energies are more affected by multiple Coulomb scattering^19^ and therefore have larger divergences. The black curve in Fig. 2 shows the average kinetic energy of the generated positrons as a function of the radial distance *r* from the propagation axis. The vertical dashed red line indicates *r*_*median*_ in Fig. 2. We find that the majority of high-energy positrons are located within *r*_*median*_. The green line in Fig. 2 illustrates the mean divergence of the positrons as a function of *r*. The divergence of a positron as it travels through the interior of lead is inversely proportional to its energy^21^. As a result, unlike high-energy positrons, most low-energy positrons are primarily distributed outside of *r*_*median*_ and have average divergences larger than 100 mrad.

**Figure 3:**
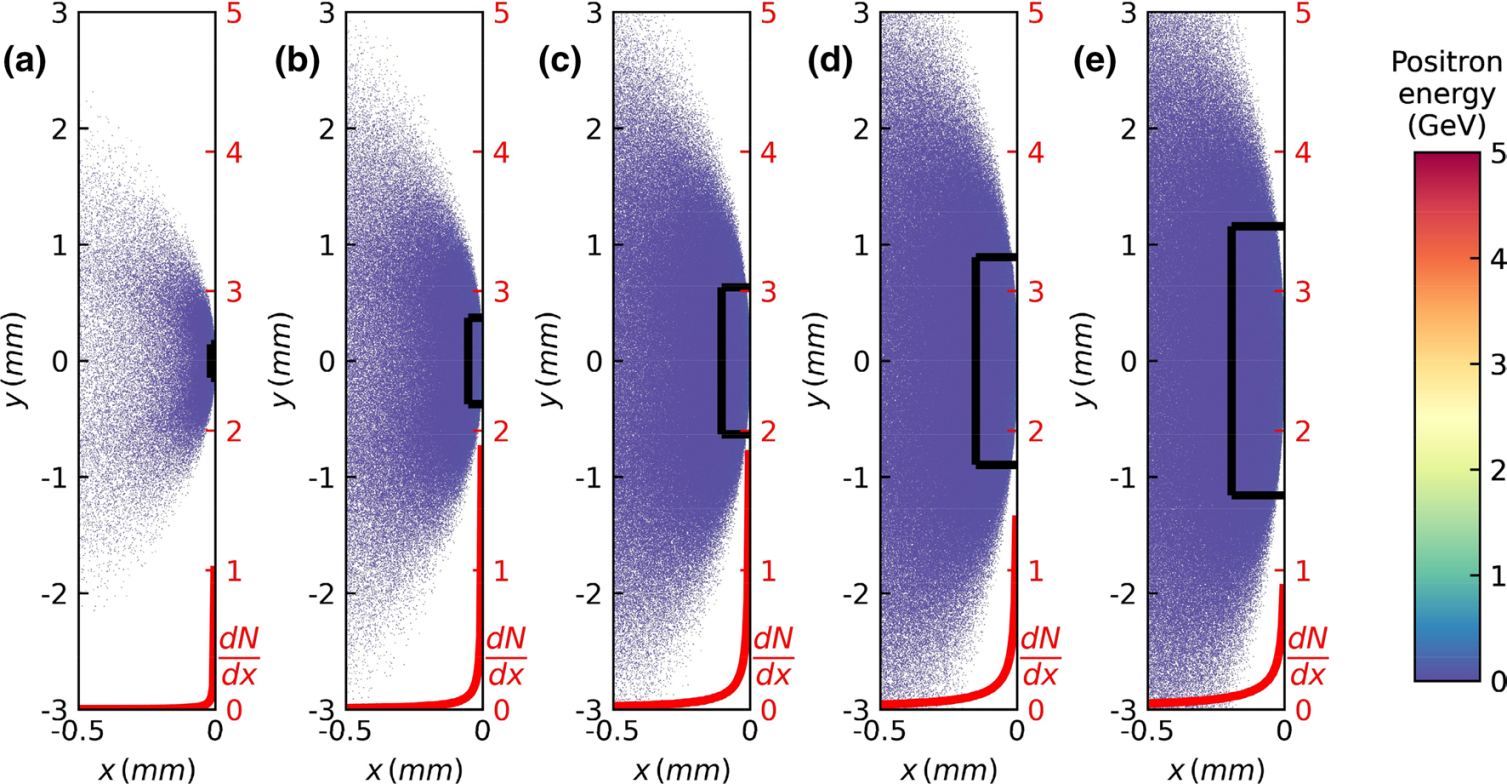
Longitudinal distribution of generated positrons for various lead converter thicknesses. The color code and the definition of the positron beam size (rectangular box in each figure) are identical to those in Fig. 1b. The driver electron energy is 5 GeV, and the lead converter thicknesses are (**a**) 1*L*_*rad*_, (**b**) 2*L*_*rad*_, (**c**) 3*L*_*rad*_ $,$(**d**) 4*L*_*rad*_, and (**e**) 5*L*_*rad*_. The red curves represent the number densities of positrons along the *x*-axis normalized by the peak value in (**a**).

Figure 3a–e illustrate the longitudinal distribution of the generated positron beam using lead converters of different thicknesses varying from *L*_*rad*_ to 5*L*_*rad*_. (See Supplementary Fig. S5 for similar results using thinner converters.) Each figure shows the spatial distribution of the positron beam generated using lead converters of different thicknesses. It is worth noting that the longitudinal thickness of the positron beam is approximately 0.2 mm after 5*L*_*rad*_ in Fig. 3e. In the same figure, we can see that the diameter of the beam is larger than 2 mm. As such, a positron beam generated from monoenergetic pencil beam electrons maintains a pancake-like shape with a small aspect ratio (< 1/10).

**Figure 4:**
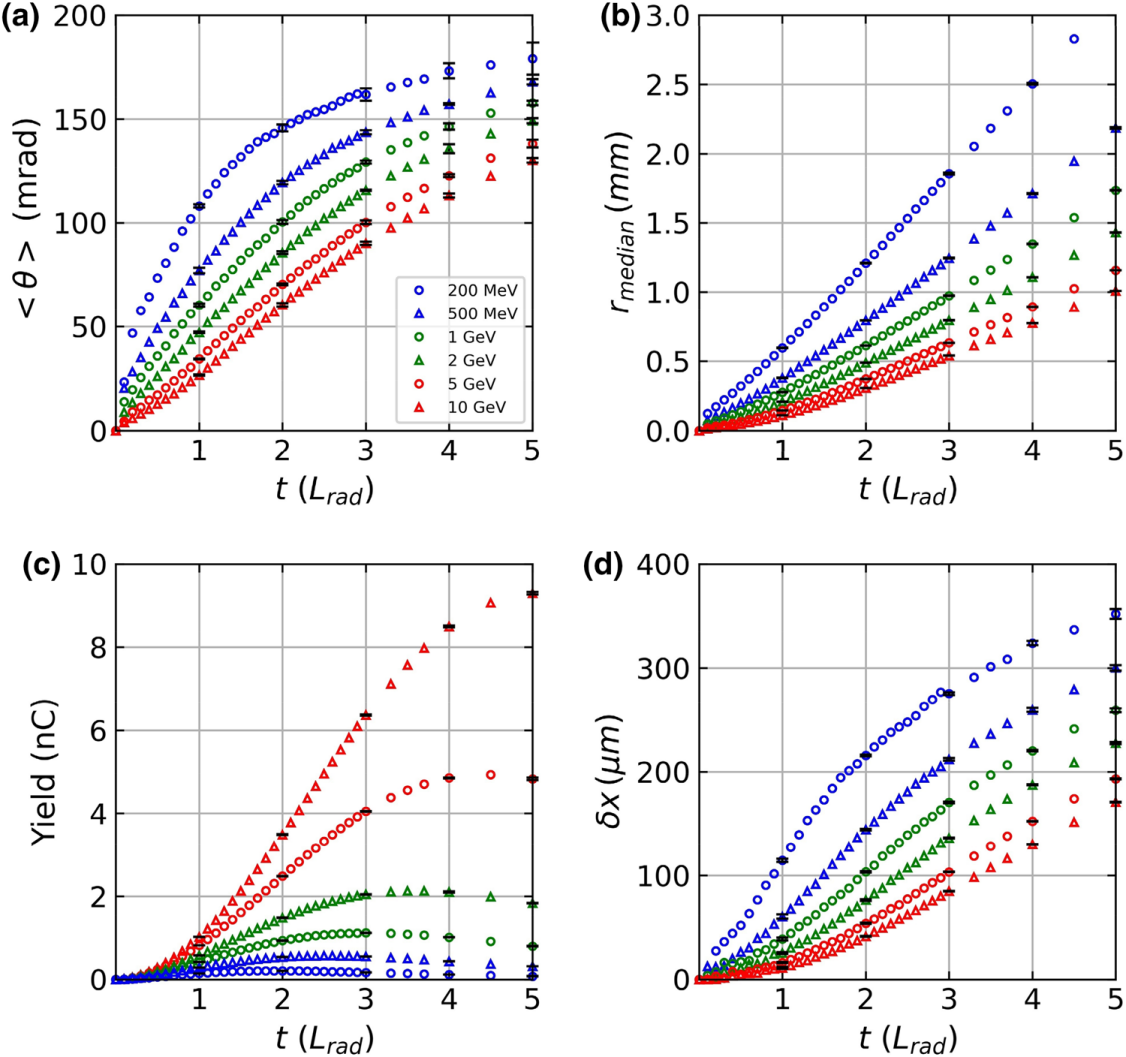
(**a**) The positron beam divergence is shown as a function of the lead converter thickness for various driver electron energies from 200 MeV to 10 GeV. (**b**) *r*_*median*_ is shown as a function of lead converter thickness for various driver electron energies. (**c**) Yield and (**d**) longitudinal thickness of the generated positron beams.

Figure 4a,b show the divergence and *r*_*median*_ of the generated positron beam, respectively, as functions of the lead converter thicknesses for different driver electron beam energies of 200 MeV, 500 MeV, 1 GeV, 2 GeV, 5 GeV, and 10 GeV. In Fig. 4a, the positron beam divergence increases with the thickness of the lead converter. For example, the positron beam divergence is larger than 110 mrad at 1*L*_*rad*_ when the driver electron energy is 200 MeV. In comparison, the beam divergence becomes less than 40 mrad at 1*L*_*rad*_ for a 5 GeV driver electron beam. Figure 4a also shows that the beam divergence becomes smaller when the driver beam energy is larger at a fixed converter thickness. In Fig. 4b, the radius of the positron beam shows a similar trend. The beam radius increases with the converter thickness, and it becomes smaller when the driver beam energy is larger at a fixed converter thickness. In Fig. 4c, the positron yield is shown as a function of lead converter thickness. The number of positrons initially increases with the converter thickness, then it saturates after a certain converter thickness. The positron yield shows a strong dependence on the driver beam energy, and the yield increases very quickly as the driver electron beam energy increases. For example, we expect a small number of positrons when a 200 MeV driver electron beam is used. In contrast, we can expect about five times more positrons (~ 5 nC) than the initial number of driver electrons (= 1 nC) at 5*L*_*rad*_ when a 5 GeV driver electron beam is used. Moreover, the thickness of the positron beam is smaller when a 5 GeV driver electron beam is used, as shown in Fig. 4d. The thickness of the positron beam is about 200 µm at 5*L*_*rad*_ when 5 GeV electrons are used, which increases to approximately 350 µm when 200 MeV electrons are employed. Increasing the energy of the driver electron beam results in the production of more positrons within a smaller volume, as shown in Fig. 4b–d. These simulation results suggest that we can achieve a high-density positron beam using a multi-GeV driver electron beam.

In order to estimate the statistical errors in our simulations, we have performed five additional simulation runs using 10^6^ initial electrons for each driver electron beam energy for the converter thicknesses of *L*_*rad*_, 2*L*_*rad*_, 3*L*_*rad*_, 4*L*_*rad*_, and 5*L*_*rad*_. In Fig. 4a–d, ± one standard deviation error bars are shown at these thicknesses. (See Supplementary Tables S1 and S2 for details.) In our simulations, the initial electron beam is assumed to be a pencil-beam. Since an actual LWFA electron beam has a divergence of a few mrad, this assumption can result in systematic errors when compared with experimental measurements. For example, Fig. 4a shows that a 5 GeV driver electron pencil-beam generates a positron beam with a divergence larger than 100 mrad after passing through more than three radiation lengths of lead. Therefore, we estimate that our assumption of a pencil-beam-like driver electron beam can cause few-percent errors in our simulation results.

In the beginning of “Positron beam structures”, we defined the beam size of the positron beam from the spatial distribution of positrons generated from a 5 GeV driver electron beam. The positron beam defined in this work reproduces some of the well-known particle physics knowledge, and Fig. 4 demonstrates the consistency of our definition even when the converter thickness and the beam driver energy are changed.

## Neutrality, density, and size

**Figure 5:**
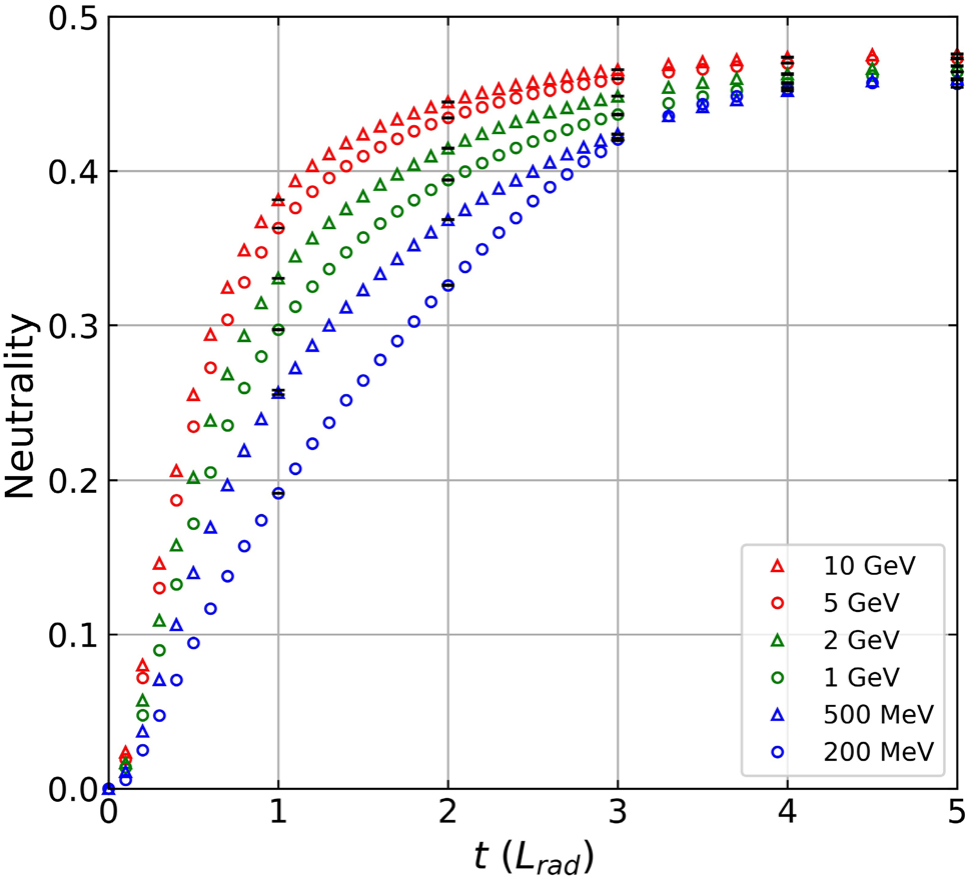
Charge neutralities of electron–positron beams, ${\mathrm{N}}_{{e}^{+}}/({\mathrm{N}}_{{e}^{-}}+{\mathrm{N}}_{{e}^{+}})$, are shown as functions of the lead converter thicknesses for different driver electron beam energies of 200 MeV, 500 MeV, 1 GeV, 2 GeV, 5 GeV, and 10 GeV.

We investigate the bulk properties of the electron–positron beam in this section. When high energy driver electrons impinge upon a thick lead converter, the majority of the initial driver electrons lose their kinetic energy via the bremsstrahlung process, producing gamma rays that can generate electron and positron pairs. This implies that the resulting electron–positron beam approaches quasi-neutrality as the beam propagates toward the end of the thick lead converter. Figure 5 shows the charge neutrality as a function of lead converter thickness at several driver electron energies from 200 MeV to 10 GeV. We calculate the charge neutrality of the electron–positron beam using ${\mathrm{N}}_{{\mathrm{e}}^{+}}/({\mathrm{N}}_{{\mathrm{e}}^{-}}+{\mathrm{N}}_{{\mathrm{e}}^{+}})$, where ${\mathrm{N}}_{{\mathrm{e}}^{-}}$ and ${\mathrm{N}}_{{\mathrm{e}}^{+}}$ are the number of electrons and positrons inside the volume defined in the previous section, respectively.

It is known that the scattering cross-section for the bremsstrahlung process increases with the energy of the driver electron beam and the scattering cross-section for the Bethe–Heitler process increases with the energy of the generated gamma ray^19^. Therefore, we expect a more efficient generation of electron–positron pairs when using a high-energy driver electron beam for a given converter thickness. Figure 5 also shows this trend at a fixed converter thickness. For example, a 5 GeV driver electron beam generates an electron–positron beam with a charge neutrality of about 0.36 at 1*L*_*rad*_, whereas the electron–positron beam has a charge neutrality less than 0.2 at 1*L*_*rad*_ when a 200 MeV driver electron beam is used.

**Figure 6:**
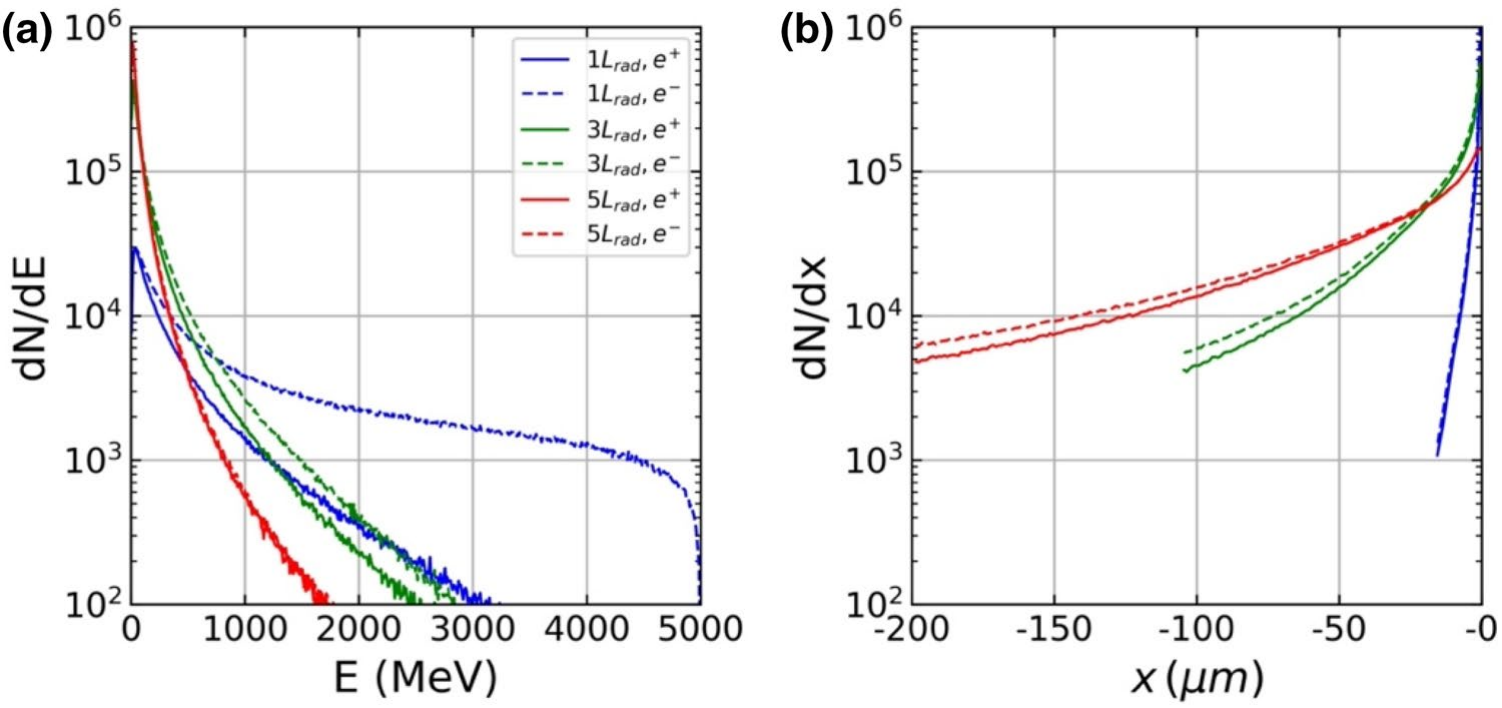
(**a**) Energy spectra of electrons (dashed lines) and positrons (solid lines) inside positron beam volume for different lead converter thicknesses. (**b**) Longitudinal distributions of electrons and positrons.

In the above charge neutrality analysis, we only compared the total number of charged particles inside the volume defined by *r*_*median*_ and δ*x*. However, in addition to the total neutrality, the energy spectra of the particles and the local neutrality of the beam should be evaluated. In Fig. 6a, the energy spectra of electrons and positrons generated within the volume are compared. With our beam size definition, the energy spectrum and the local neutrality exhibited predictable behavior of electromagnetic shower as the lad thickness increased. At 5*L*_*rad*_, the energy spectra of electrons and positrons are almost identical. On the other hand, the energy spectrum of electrons and that of positrons differ significantly at 1*L*_*rad*_ in Fig. 6a. We see that there is a much smaller number of positrons at high energies. This is because there was insufficient time for the electromagnetic shower to evolve. There are more high-energy electrons than positrons because there are many primary electrons that did not interact yet. As the thickness increases, nearly all the primary electrons interact and the energy distributions of electrons and positrons become comparable. The difference between the number densities of electrons and positrons along the longitudinal axis is also small at 5*L*_*rad*_, which can be seen from the dashed red line and the solid red line in Fig. 6b, respectively.

**Figure 7:**
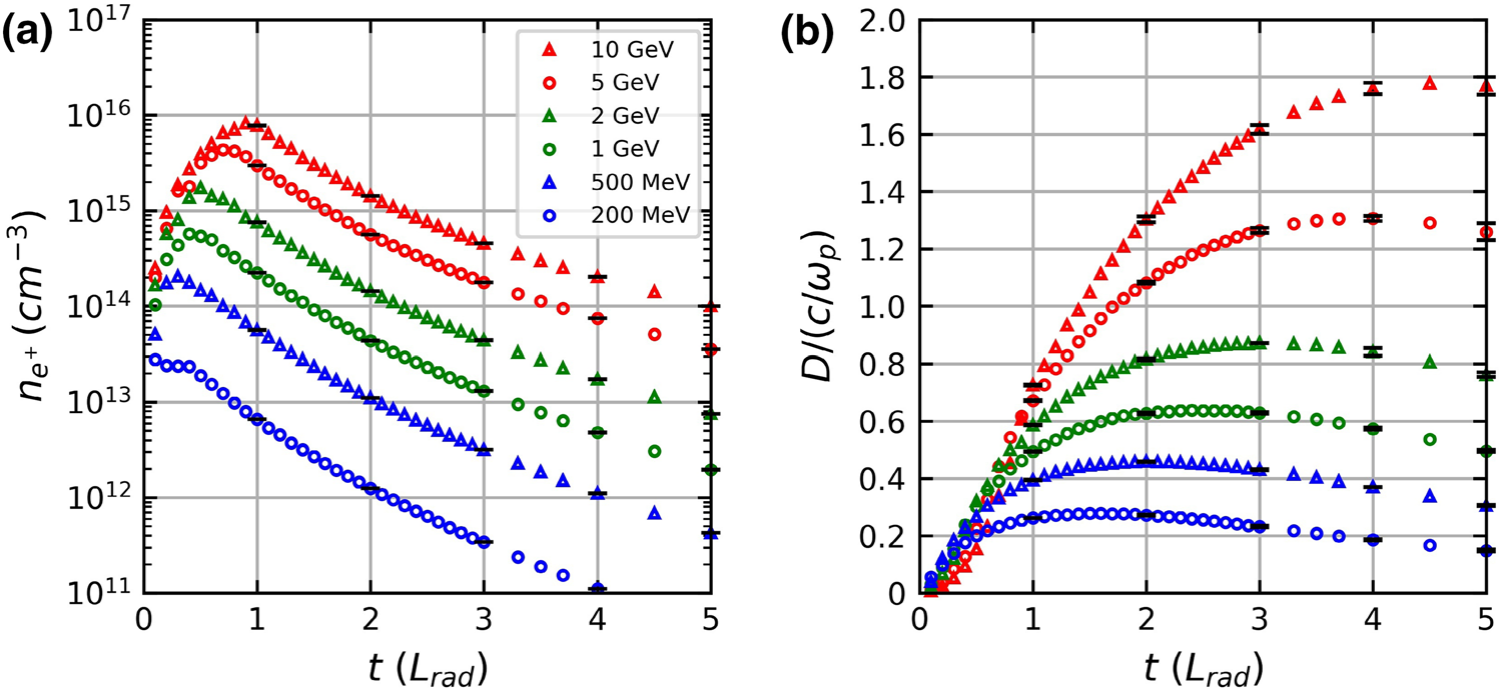
(**a**) Densities of positron beams are shown as functions of the lead converter thicknesses for different driver electron beam energies of 200 MeV, 500 MeV, 1 GeV, 2 GeV, 5 GeV, and 10 GeV. (**b**) *D/(c*/ω_p_) values of the positron beams are shown as functions of the lead converter thicknesses for different driver electron beam energies from 200 MeV to 10 GeV.

In Fig. 7a,b, we investigate the number density of the generated positron beam and the conditions for the beam to be called a pair plasma. Thus far, we have assumed the driver electron beam to be a pencil beam. However, when calculating the number density of positrons, it is necessary to consider the divergence and size of the driver LWFA electron beam. The typical size^22^ of an LWFA electron beam in the longitudinal direction is approximately 10 μm. Thus, the net thickness of the generated positron beam is approximated as (10 + δ*x*) μm in our calculations. In addition, we use a typical LWFA electron beam radius, 100 μm, as the radius of the positron beam when the *r*_*median*_ of the generated positron beam is smaller than 100 μm. Further, the number of positrons within *r*_*median*_ and the longitudinal position within δ*x* is considered when calculating the positron number density. The number density in Fig. 7a is calculated assuming that the driver electron beam has a charge of 1 nC. This assumption is based on a recent LWFA electron beam experiment that demonstrated the generation of a GeV electron beam with a charge of several hundred pC^13^. The solid red line in Fig. 7a shows the positron number density of about $5\times {10}^{15}$ cm^−3^ at 1*L*_*rad*_ of lead converter. As reported by Williams et al*.*^20,23,24^, when using a 200 MeV driver electron beam the number density of the positron beam decreases as the converter thickness increases. However, when the driver electrons have energies above 200 MeV, the positron number density increases initially and reaches a peak within one radiation length. This seems to be consistent with Fig. 4, where the radius and thickness of the positron beam increase with the converter thickness but the increase in positron yield is saturated.

To observe the plasma phenomenon using an electron–positron beam, the size of the generated beam must be greater than the length of the plasma interaction with the electromagnetic field^8^. Skin depth is the length at which plasma interacts with electromagnetic waves. For example, to observe electromagnetic wave instability such as Weibel instability, the size of the pair beam must be larger than the skin depth^8^. The size of the generated positron beam, *D* = 2*r*_*median*_, is divided by its relativistic plasma skin depth, *c*/ω_p_, as depicted in Fig. 7b. Since the generated electron–positron beams are relativistic in our simulations, we used the relativistic plasma skin depth, $c/{\omega }_{p}=c/\sqrt{\gamma m{\epsilon }_{0}/n{e}^{2}}$, where *m* is the mass of the electron, *ε*_0_ is the electric permittivity, *n* is the number density of the electron–positron pair, and *e* is the electric charge of the electron. For relativistic gamma, *γ* $,$ we used the relativistic gamma of the mean longitudinal velocity of the positron beam. In Fig. 7b, the *D*/(*c*/*ω*_p_) value exceeds 1.0 if the lead converter is thicker than 1.0 radiation length for a 10 GeV driver electron beam. For a 5 GeV driver electron beam, the ratio becomes larger than 1.0 when the lead converter is thicker than 1.3 radiation lengths. In the case of a 2 GeV driver electron beam, the ratio is slightly smaller than 1.0. In summary, Fig. 7b shows that a multi-GeV driver electron beam can generate a positron beam whose diameter is larger than the skin depth, suggesting the possibility of showing the collective behavior of a plasma. Since Figs. 4, 5, and 7a show that a multi-GeV driver electron beam can generate a dense quasi-neutral (neutrality > 0.4) electron–positron beam, the resulting high-density electron–positron beam can now be regarded as a pair plasma.

## Conclusion

We investigated the characteristics of the electron–positron beam generated by a driver electron beam with energies ranging from 200 MeV to 10 GeV using a lead converter of varying thicknesses from 0.1*L*_*rad*_ to 5*L*_*rad*_. Irradiating the converter with monoenergetic pencil beam electrons resulted in the generation of a positron beam that maintained a pancake-like structure while showering into the lead converter. The electron–positron beam produced with a 1 nC, 5 GeV driver electron beam has a quasi-neutral property and a high average positron density of 10^15^–10^16^ cm^−3^ at lead converter thicknesses of 1–2 radiation lengths. The results show that a high-density quasi-neutral electron–positron plasma with a size greater than the relativistic skin depth can be produced in a laboratory using a 1 nC, 5–10 GeV electron beam. Our study will provide a valuable guide in interpreting the physical properties of experimentally generated laser-driven electron–positron beams.

## Supplementary Information

Supplementary Information.

## Data Availability

The data that support the findings of this study are available from the corresponding author upon reasonable request.
