# Supplementary material for: Characterization of relativistic electron–positron beams produced with laser-accelerated GeV electrons
Source: Sci Rep. 2023 Jan 6;13:310. doi: 10.1038/s41598-023-27617-0 (PMC9823095; doi:10.1038/s41598-023-27617-0)
Supplement: Supplementary file 1 — Supplementary Information. [file 41598_2023_27617_MOESM1_ESM.docx]

Characterization of relativistic electron-positron beams produced with laser-accelerated GeV electrons

**Hoon Song,^1, 2^ Chul Min Kim,^1, 3^ Junho Won,^1, 2^ Jaehyun Song,^1, 2^ Seongmin Lee,^1, 2^ Chang-Mo Ryu,^1^ Woosuk Bang,^1, 2, *^ and Chang Hee Nam^1, 2^**

*^1)^Center for Relativistic Laser Science, Institute for Basic Science (IBS), Gwangju 61005, Korea*

*^2)^Department of Physics and Photon Science, GIST, 61005, Korea*

*^3)^Advanced Photonics Research Institute, GIST, Gwangju 61005, Korea*

1. **Statistical error analysis of the positron beam parameters**

We defined the volume of the positron beam as a cylindrical volume determined by *r_median_* and *δ_x_*. Other beam parameters, such as divergence, yield, density, and neutrality, were calculated based on this definition. Each data point in Figs. 4, 5, and 7 corresponds to a single simulation run using 10^6^ initial electrons. In this section, we provide the relative standard deviations (RSD (%) = (standard deviation/mean) ×100%) of these beam parameters after performing five additional simulation runs using 10^6^ initial electrons at each driver electron beam energy for converter thicknesses of *L_rad_,* 2*L_rad_,* 3*L_rad_,* 4*L_rad_,* and 5*L_rad_*. Supplementary Tables S1 and S2 show the errors for the initial driver electron beam energy of 1 GeV and 5 GeV, respectively. The beam parameters were calculated after performing five identical simulation runs with different random seed and post-processed electrons and positrons.

| $\mathrm{RSD} (\%)$ | $1L_{rad}$ | $2L_{rad}$ | $3L_{rad}$ | $4L_{rad}$ | $5L_{rad}$ |
| --- | --- | --- | --- | --- | --- |
| $<\theta>$ | *0.60* | *0.42* | *0.27* | *0.59* | *0.34* |
| $r_{median}$ | *0.28* | *0.04* | *0.09* | *0.07* | *0.06* |
| $\mathrm{Yield}$ | *0.23* | *0.15* | *0.15* | *0.22* | *0.24* |
| $\delta_{x}$ | *1.78* | *0.35* | *0.23* | *0.15* | *0.35* |
| Neutrality | *0.18* | *0.10* | *0.15* | *0.15* | *0.13* |
| Density | *1.66* | *0.36* | *0.40* | *0.25* | *0.24* |
| $D/(c/\omega_{p})$ | *0.58* | *0.19* | *0.20* | *0.28* | *0.18* |

Supplementary Table S1. Relative standard deviations of positron beam parameters for a 1 GeV driver electron beam at converter thicknesses of *L_rad_*, 2*L_rad_*, 3*L_rad_*, 4*L_rad_*, and 5*L_rad_*. The errors were calculated from five additional simulation runs using 10^6^ initial electrons.

| $\mathrm{RSD}(\%)$ | $1L_{rad}$ | $2L_{rad}$ | $3L_{rad}$ | $4L_{rad}$ | $5L_{rad}$ |
| --- | --- | --- | --- | --- | --- |
| $<\theta>$ | *0.30* | *0.30* | *0.45* | *0.26* | *0.66* |
| $r_{median}$ | *0.11* | *0.10* | *0.06* | *0.02* | *0.02* |
| $\mathrm{Yield}$ | *0.09* | *0.07* | *0.11* | *0.19* | *0.3* |
| $\delta_{x}$ | *1.60* | *0.60* | *0.08* | *0.07* | *0.14* |
| Neutrality | *0.05* | *0.06* | *0.05* | *0.06* | *0.18* |
| Density | *1.71* | *0.73* | *0.14* | *0.17* | *0.24* |
| $D/(c/\omega_{p})$ | *0.67* | *0.32* | *0.23* | *0.17* | *0.39* |

**Supplementary Table S2.** Relative standard deviations of positron beam parameters for a 5 GeV driver electron beam at converter thicknesses of *L_rad_*, 2*L_rad_*, 3*L_rad_*, 4*L_rad_*, and 5*L_rad_*. The errors were calculated from five additional simulation runs using 10^6^ initial electrons.

1. **Visualization of the spatial distribution of positrons**

In Fig. 1(b), an arc structure is visible from the spatial distribution of the generated positrons. In this section, we examine the same spatial distribution data using different color scales for the positron energy. Supplementary Figs. S1(a) and S1(b) show that high energy (> 200 MeV) positrons are distributed around the propagation axis and near the front surface of the positron beam, respectively. Low energy positrons are spread out mostly outside the cylindrical volume. Supplementary Figs. S1(b), S1(d), and S1(f) suggest that low energy (< 50 MeV) positrons with large divergence angles form the arc-shaped structure seen in Fig. 1(b).


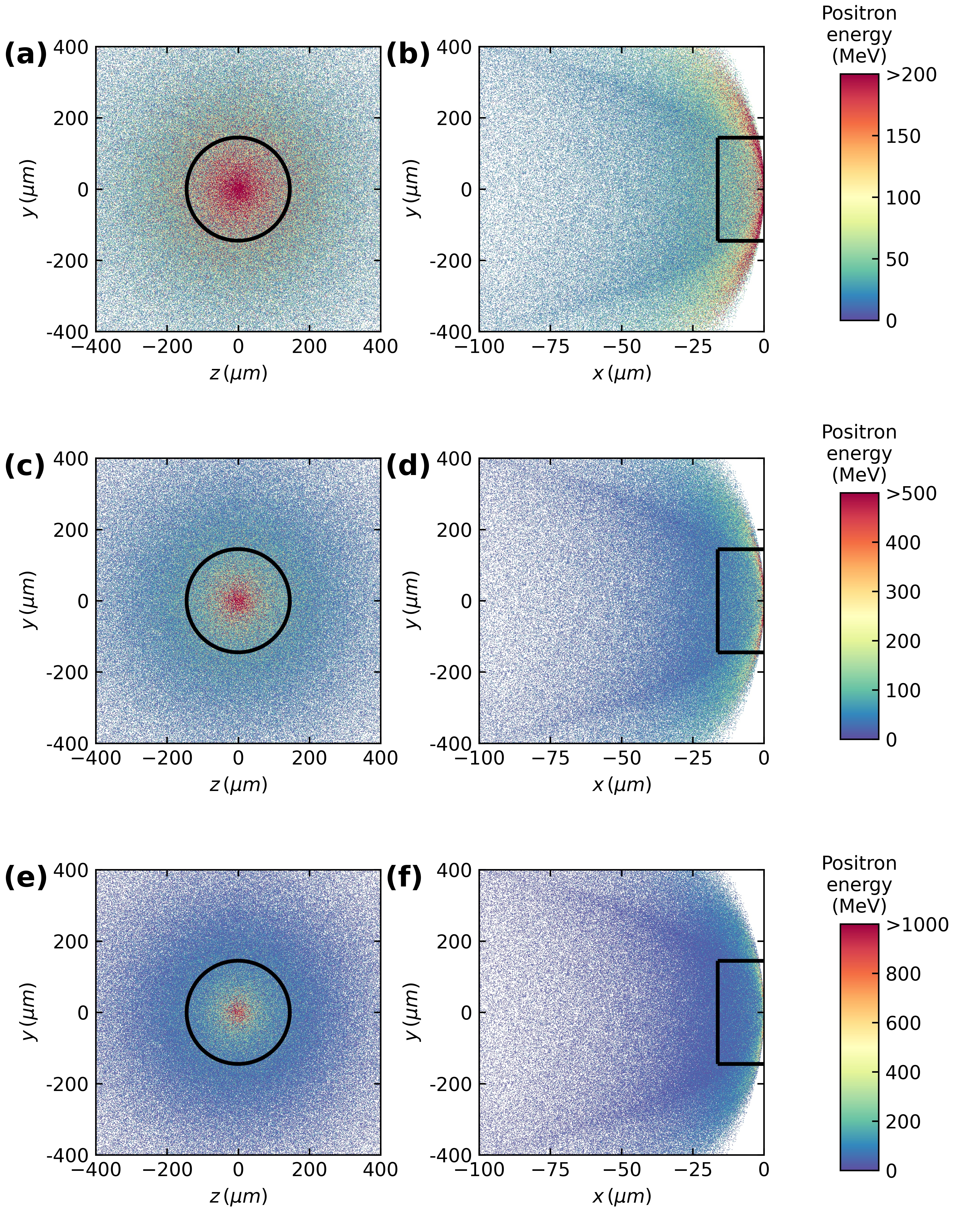


**Supplementary Fig. S1.** Spatial distribution of generated positrons. (a), (c), and (e) Transversal distribution of the generated positrons using different color scales for the positron energy. (b), (d), and (e) Longitudinal distribution of the generated positrons using different color scales. Each row presents the identical positron population with a different energy color scale. The driver electron energy is 5 GeV, and the lead converter thickness is *L_rad_.*

Supplementary Fig. S2 shows the spatial distribution of the generated positrons in four different energy ranges. For example, Supplementary Figs. S2(a) and (b) show the transversal and longitudinal distributions of the positrons, respectively, with kinetic energy from 1 MeV to 50 MeV. Supplementary Fig. S2(b) clearly shows the arc structure seen in Fig. 1(b). Apparently, there are sufficiently many low-energy positrons with large divergence angles to form a visible arc structure. On the other hand, positrons having kinetic energy larger than 50 MeV are plotted in Supplementary Figs. S2(c)–(h), and they do not exhibit the observed arc structure.


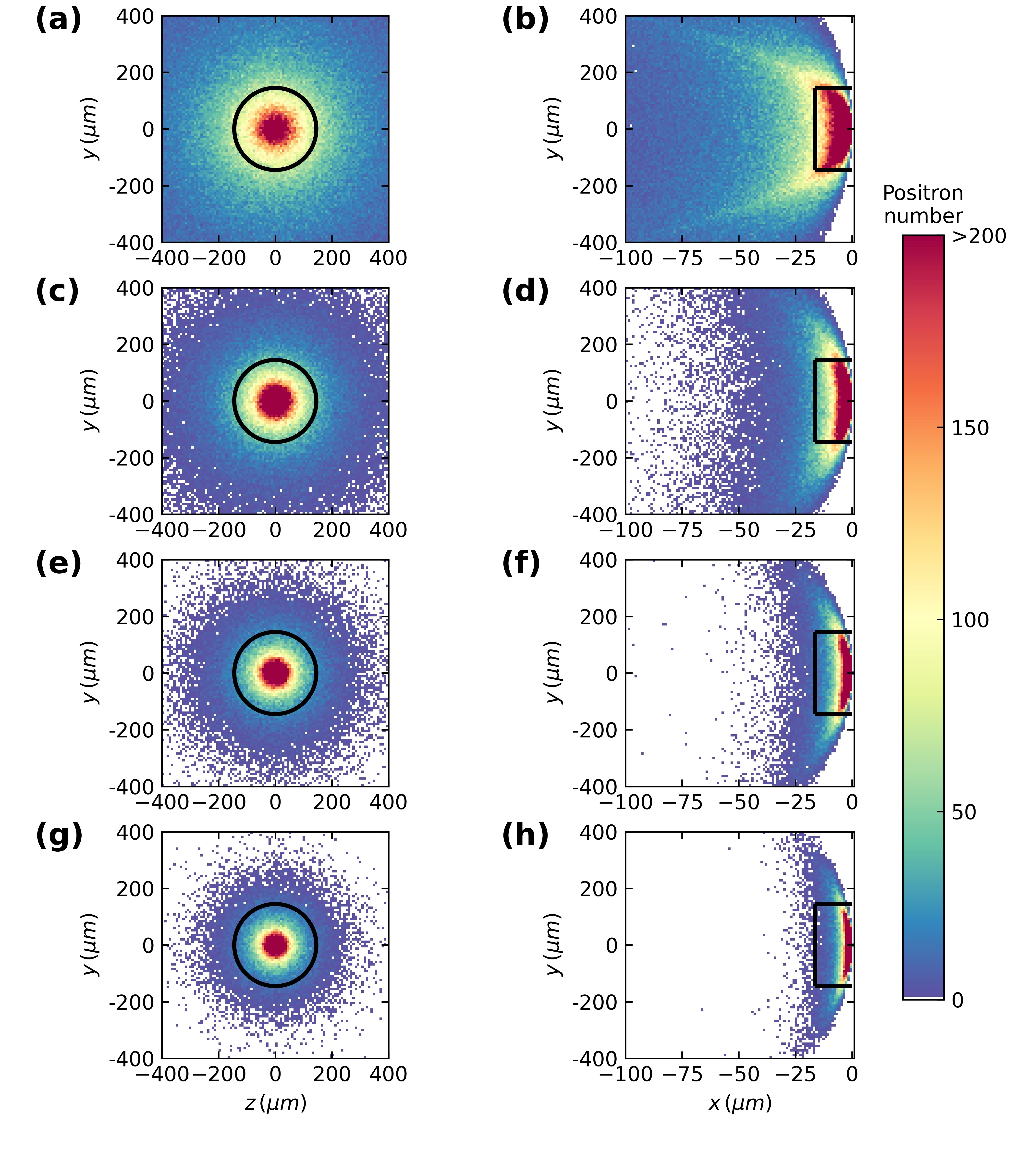


**Supplementary Fig. S2.** Spatial distribution of the generated positrons in the energy range of (a, b) 1–50 MeV, (c, d) 50–100 MeV, (e, f) 100–150 MeV, and (g, h) 150–200 MeV. The left columns show the transversal distribution of positrons within a cell area of $dz\times dy=8 \mu m\times8 \mu m$. The right columns illustrate the longitudinal distribution of positrons within a cell area of $dx\times dy=1 \mu m\times8 \mu m$. The color of each cell represents the number of positrons in the cell from 0 (white) to >200 (red, saturated). The driver electron energy is 5 GeV, and the lead converter thickness is *L_rad_.*

The spatial distributions of the generated positrons are shown in Supplementary Figs. S3(a), (b). The color of each cell represents the number of positrons in the cell in log scale. It is evident from Supplementary Fig. S3(b) that the generated positrons are highly concentrated within the rectangular box, justifying our definition of the positron beam. An arc structure is also visible in this figure.


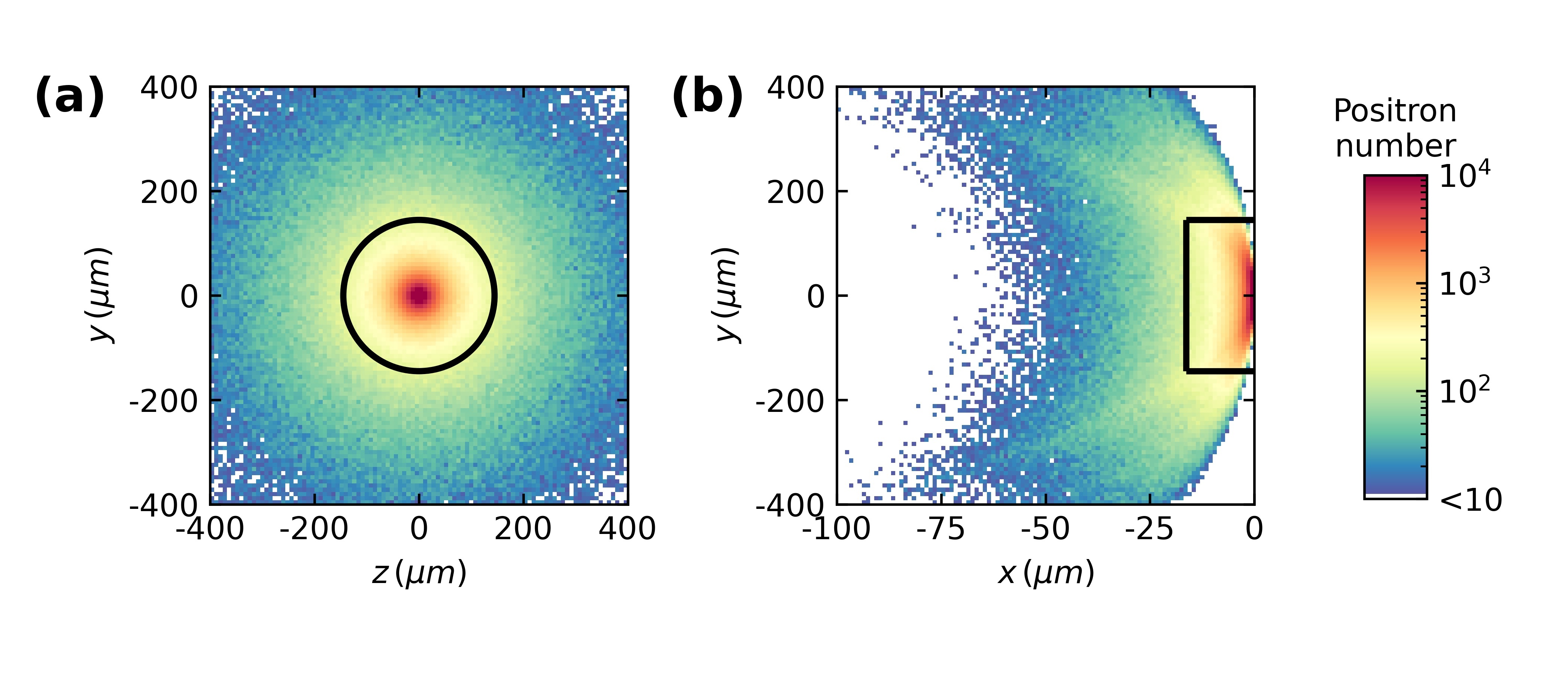


**Supplementary Fig S3.** (a) Transversal distribution of the generated positrons. The color of each cell represents the number of positrons in log scale from 10 (blue) to 104 (red). The cell area is *dz×dy*=8 μm×8 μm. (b) Longitudinal distribution of the generated positrons. The cell area is *dx×dy*=1 μm×8 μm. Again, the black rectangle represents the dimensions of the positron beam.

In Supplementary Fig. S4, the radial distribution of the generated positrons, *dN/dr*, is shown as a function of r from 0 to 400 μm. The radius of the positron beam, *r_median_*, is also shown as a dashed line in the plot.


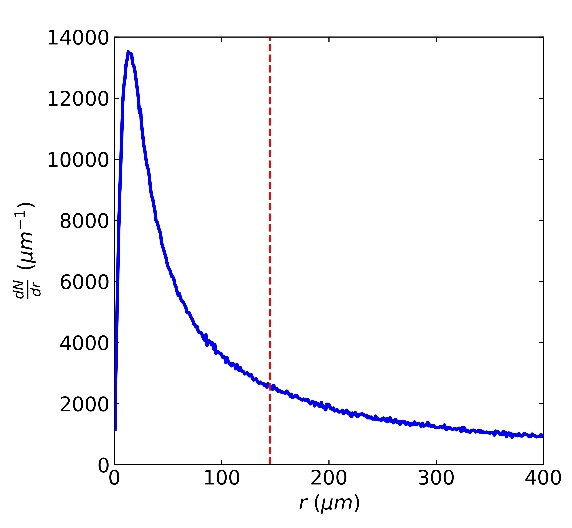


**Supplementary Fig. S4** Radial distribution function ($dN/dr (\mu m^{-1}))$ is shown. The dashed red line represents *r_median_*=145 $\mu m$.

1. Longitudinal distribution of positrons for thin converters

Figure 3 shows the longitudinal distribution of the positron beam for converter thicknesses of *L_rad_*, 2*L_rad_*, 3*L_rad_*, 4*L_rad_*, and 5*L_rad_*. In this section, we present the longitudinal distribution of the generated positron beam after thin converters of thicknesses of 0.1*L_rad_*, 0.3*L_rad_*, 0.5*L_rad_*, 0.7*L_rad_*, and 0.9*L_rad_*.


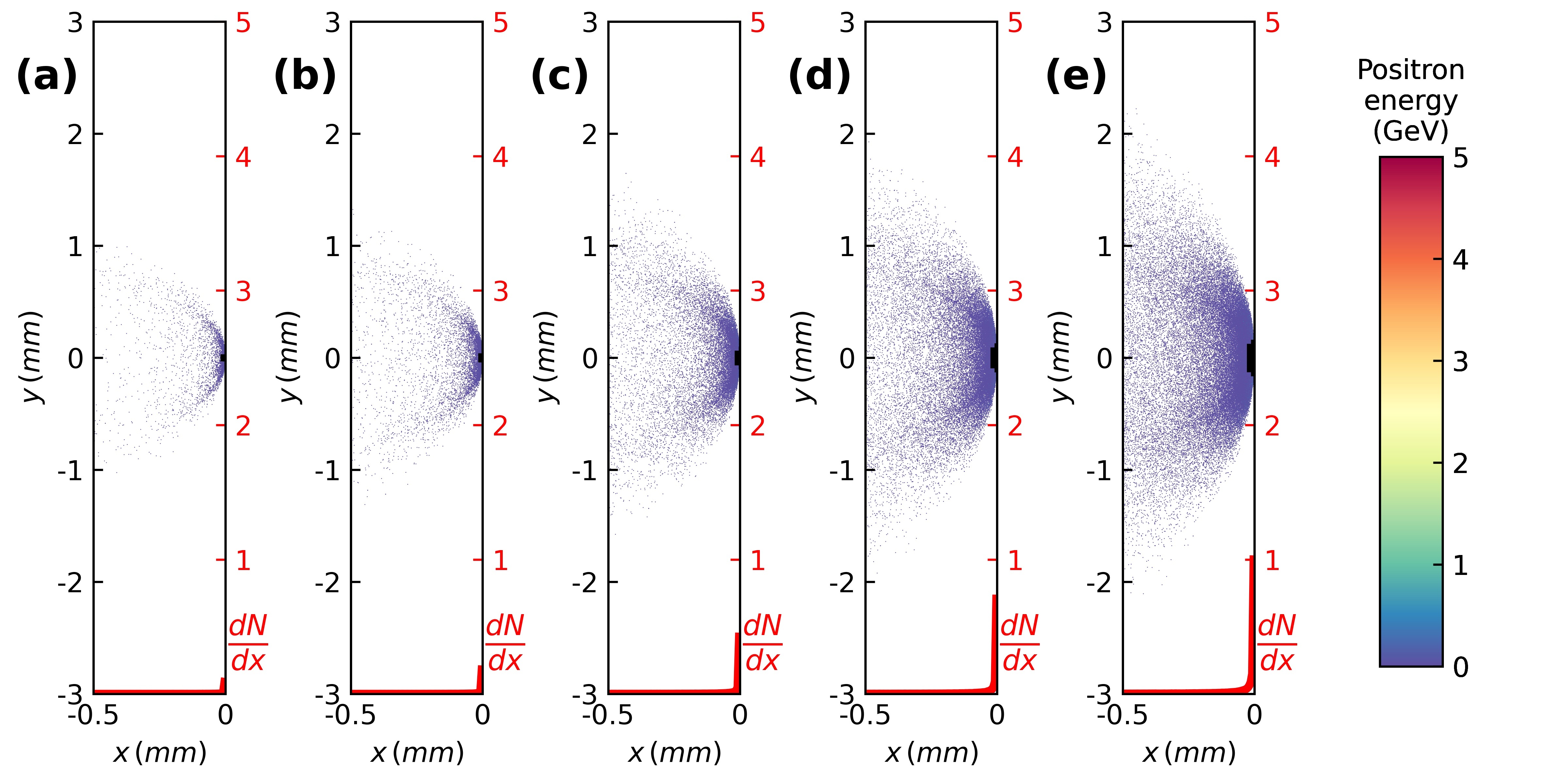


**Supplementary Fig. S5.** Longitudinal distribution of the generated positrons using various lead converter thicknesses. The lead converter thicknesses are (a) 0.1*L_rad_*, (b) 0.3*L_rad_*,(c) 0.5*L_rad_*,(d) 0.7*L_rad_*, and (e) 0.9*L_rad_*. The red curves represent the number densities of positrons along the x-axis normalized by the peak value in Fig. 3(a).
